# Interferon: A Sharp Sword to Overcome HCV or HBV-Related Liver Diseases?

**Published:** 2010-09-01

**Authors:** Wei-Dong Jia, Chuan-Hai Zhang

**Affiliations:** 1 Department of Hepatic Surgery, Anhui Provincial Hospital, Anhui Medical University, Anhui, China; 2 Centre for the Study of Liver Cancer, Anhui Provincial Hospital, Anhui Medical University, Anhui, China

Dear Editor,

We read with great interest the article entitled "The Comparative Efficacy and Safety of Peginterferon Alpha-2a vs. 2b for the Treatment of Chronic HCV Infection: A Meta-Analysis" by Alavian et al. [1]. published in Hepatitis Monthly. In this article, the authors performed an elegant meta-analysis of randomized controlled trials to compare the different effects (including efficacy and safety) of using two types of peginterferon, alpha-2a (PEG-IFN-α2a) and 2b (PEG-IFN-α2b)  in the treatment of chronic hepatitis C virus (HCV) infection. By pooling data from 7 randomized controlled trials, the authors concluded that PEG-IFN-α2a with similar safety is more effective than PEG-IFN-α2b; and that a longer duration of maximum serum concentration compared with PEG-IFN-α2b yields a greater sustained virological response and higher neutropenia in PEG-IFN-α 2a recipients. These analysis results are of important clinical directive significance for using interferon alpha (IFN-α) to treat HCV-related liver diseases.

HCV and HBV infections are major global causes of liver-related morbidity and mortality; and they are also the most common etiologies of hepatocellular carcinoma (HCC). However, IFN-α not only has antiviral activities with clearance or suppression of HBV and HCV, but also possesses anti-tumor properties including antiproliferative, antiangiogenic, and immunomodulatory effects [2][3][4]. In recent years, we and others have also done some excellent research work on this issue. In 2006 [5], we performed an immunohistochemical study of P48 staining on specimens that were collected from patients, in a randomized trial, who received postoperative IFN-a therapy (80 patients) and who did not receive postoperative IFN-a therapy (75 patients); study results indicated that P48 was useful as a predictive marker of outcome after postoperative IFN-a treatment in patients with HBV-related HCC. Owing to the fact that the included subpopulation in our study consisted mostly of HBV-positive patients, whether our study results were suitable for HCV-positive patients is still unknown; so our further research may focus on this subpopulation.

To the best of our knowledge, IFN-α has been widely used in the prevention and treatment of HCV or HBV- related liver diseases (including hepatitis, cirrhosis, HCC etc.) both in western and Asian countries; it alone or in combination with other agents might offer a good therapeutic option for patients with HCV or HBV-related liver diseases. Further explorations of the benefits of this use would be worthwhile.
